# Hypofractionated Whole Breast Irradiation and Boost-IOERT in Early Stage Breast Cancer (HIOB): First Clinical Results of a Prospective Multicenter Trial (NCT01343459)

**DOI:** 10.3390/cancers14061396

**Published:** 2022-03-09

**Authors:** Gerd Fastner, Roland Reitsamer, Christoph Gaisberger, Wolfgang Hitzl, Bartosz Urbański, Dawid Murawa, Christiane Matuschek, Wilfried Budach, Antonella Ciabattoni, Juliann Reiland, Marie Molnar, Cristiana Vidali, Claudia Schumacher, Felix Sedlmayer, on behalf of the HIOB Trialist Group

**Affiliations:** 1 Department of Radiotherapy and Radio-Oncology, Paracelsus Medical University, University Hospital Salzburg, Landeskrankenhaus, 5020 Salzburg, Austria; c.gaisberger@salk.at (C.G.); f.sedlmayer@salk.at (F.S.); 2 Department of Gynecology, Paracelsus Medical University, University Hospital Salzburg, Landeskrankenhaus, 5020 Salzburg, Austria; r.reitsamer@salk.at; 3 Research Office—Biostatistics, Paracelsus Medical University, 5020 Salzburg, Austria; wolfgang.hitzl@pmu.ac.at; 4 Department of Ophthalmology and Optometry, Paracelsus Medical University, University Hospital Salzburg, Landeskrankenhaus, 5020 Salzburg, Austria; 5 Research Program for Experimental Ophthalmology and Glaucoma Research, Paracelsus Medical University, 5020 Salzburg, Austria; 6 Department of Radiotherapy and Gynecological Oncology, Greater Poland Cancer Centre, 61-866 Poznań, Poland; bartosz.urbanski@wco.pl; 7 Clinic of General Surgery and Surgical Oncology, Faculty of Medicine and Health Sciences, University of Zielona Góra, 65-046 Zielona Gora, Poland; dawid.murawa@skpp.edu.pl; 8 Clinic of Surgical Oncology, University of Medical Sciences, 61-701 Poznan, Poland; 9 Department of Radiation Oncology, Medical Faculty, Heinrich Heine University, 40225 Düsseldorf, Germany; matuschek@med.uni-duesseldorf.de (C.M.); wilfried.budach@med.uni-duesseldorf.de (W.B.); 10 U.O.C. Radioterapia, San Filippo Neri Hospital, ASL Roma 1, 00135 Rome, Italy; antonella.ciabattoni@as1roma1.it; 11 Avera McKennan Hospitals and University Health System, Avera Medical Group Comprehensive Breast Care, Sioux Falls, SD 57105, USA; juliann.reiland@gmail.com; 12 Department of Radiotherapy and Radiooncology, Landeskrankenhaus Klagenfurt, 9020 Klagenfurt, Austria; marie.molnar@lkh-klu.at; 13 Department of Radiation Oncology, Azienda Sanitaria Universitaria Integrata di Trieste, 34128 Trieste, Italy; cristiana.vidali@libero.it; 14 Breast Center, Department of Senology, St. Elisabeth Hospital Cologne-Hohenlind, 50935 Cologne, Germany; claudia.schumacher@hohenlind.de

**Keywords:** hypofractionation, whole breast irradiation, intraoperative radiation therapy, boost, electrons, IOERT, cosmesis, toxicity

## Abstract

**Simple Summary:**

Intraoperative electron radiation therapy (IOERT) has repeatedly demonstrated its power to yield high local control rates in all risk constellation of patients with invasive breast cancer when given as tumor bed boost prior to conventional whole breast irradiation (WBI) after breast conserving surgery (BCS). Since the standard for WBI continuously moved towards hypofractionation, we initiated a prospective trial in 2011 where we combined a high precision IOERT boost with a “moderate” hypofractionation schedule (15 × 2.7 Gy), the HIOB-trial [NCT01343459], as clinical evidence for this combination was scarce. Our results demonstrated a low five-year local recurrence rate (overall two events in a population of 1119 patients), which undershot such best evidences in two age groups (41–50 y and >50 y). As acute and late toxicity were mild with no impaired cosmetic outcome, the HIOB-concept seems to be a viable treatment option for patients who underwent BCS and intended WBI.

**Abstract:**

Background and purpose: To investigate intraoperative electron radiation therapy (IOERT) as a tumor bed boost during breast conserving surgery (BCS) followed by hypofractionated whole breast irradiation (HWBI) on age-correlated in-breast recurrence (IBR) rates in patients with low- to high-risk invasive breast cancer. Material and methods: BCS and IOERT (11.1 Gy) preceded a HWBI (40.5 Gy) in 15 fractions. Five-year IBR-rates were compared by a sequential ratio test (SQRT) with best evidences in three age groups (35–40 y and 41–50 y: 3.6%, >50 y: 2%) in a prospective single arm design. Null hypothesis (H0) was defined to undershoot these benchmarks for proof of superiority. Results: Of 1445 enrolled patients, 326 met exclusion criteria, leaving 1119 as eligible for analysis. After a median follow-up of 50 months (range 0.7–104), we detected two local recurrences, both in the age group >50 y. With no observed IBR, superiority was demonstrated for the patient groups 41–50 and >50 y, respectively. For the youngest group (35–40 y), no appropriate statistical evaluation was yet possible due to insufficient recruitment. Conclusions: In terms of five-year IBR-rates, Boost-IOERT followed by HWBI has been demonstrated to be superior in patients older than 50 and in the age group 41–50 when compared to best published evidence until 2010.

## 1. Introduction

Moderate hypofractionated whole breast irradiation (HWBI) of invasive breast cancer after breast conserving surgery (BCS) has been established as standard of care by numerous prospective trials providing also long-term observation [1]. In these trials, HWBI with moderate fractional sizes of 2.6–3.2 Gy up to total doses of 39–40 Gy in three weeks demonstrated non-inferiority compared to normofractionation in terms of local control, survival, late toxicity, cosmetic outcome (CO), and quality of life [1]. In addition, a tumor bed boost of 10–16 Gy has been described as further decreasing in breast recurrence (IBR) rates, independent of the patient’s age [2], but with the highest detectable benefit for women <40 years [3]. Aside from younger age, further indications for a subsequent tumor bed boost are well summarized in diverse guidelines [4,5,6,7] and include: tumor grade 3, tumor size >2 cm, biological cancer subtypes at higher-risk (e.g., triple negativity or positivity for Her2-neu), predominant ductal carcinoma in situ (DCIS) components, and R1-status. As a consequence, attempts were made to treat patients with a higher IBR-risk with escalated tumor bed boost doses, either with external, interstitial, or intraoperative techniques (IORT) with 50-kv X-rays or electrons (IOERT) [8,9,10,11,12]. In general and from a radiooncological point of view, IORT provide several advantages like small and precise target volumes with complete skin protection as a prerequisite for an avoidance of geographic misses, but a better cosmetic outcome while shortening the overall treatment time, respectively [13,14]. Furthermore, the biological effects of high-single doses on tumor-cell kill have been published several times during the last decades [15,16,17,18,19,20,21]. IOERT as anticipated tumorbed boost, was established two decades ago [10], which led to favorable local control rates in several risk constellations [13]. A direct comparison of IOERT versus external electrons as sequential tumorbed-boost in patients with early breast cancer stages I/II, demonstrated a significant superiority of the intraoperative approach in terms of local control after five years of follow-up [22,23]. However, this effect was not observed within a randomized phase III trial after a median observation of ten years, but with a clear delay for the first occurrence of local recurrences after IOERT [23]. The same question investigates an ongoing prospective randomized trial for intraoperative photons (TARGIT-B), which started in 2013 (ClinicalTrials.gov Identifier: NCT01792726). An estimated enrollment of 1796 young (<46 years) or high-risk participants were scheduled until 2022 with local control as primary and survival, treatment tolerance as well as quality of life as secondary endpoints. Nonetheless, probable gain of a dose augmentation to the tumor bed combined with HWBI has not been explicitly addressed by the respective prospective trials, especially not in direct correlation with age. This prompted the International Society of Intraoperative Radiation Therapy (ISIORT) to initiate a prospective multicenter trial (HIOB ClinicalTrial.gov NCT01343459, accessed on 27 April 2011) in 2011. Within this study, we investigated the outcome in patients treated by an IOERT-boost during BCS followed by HWBI. Primary endpoint was defined as comparison of observed five-year IBR-rates against best published results of randomized controlled trials (RCTs) for three different age groups (35–40, 41–50, and >50 y). Results on treatment tolerance and cosmetic outcome were published previously [24], the present manuscript reports on the first oncological results.

## 2. Material and Methods

### 2.1. Study Patients

Patients 35 years of age or older with histologically confirmed invasive breast cancer, were eligible for the trial, with no further restrictions for biological subtypes, tumor stages of pT1–2, breast planning target volumes (PTV) of ≤2500 mL, R0-resection and no subsequent re-excision after IOERT, and pN0–1 provided no indication for regional node irradiation (RNI) was given. There were also no set limits towards adjuvant or neoadjuvant systemic treatment (protocol amendment 21 August 2015). Table 1 and Supplementary Table S1 illustrate patient characteristics and exclusion criteria, respectively. The trial was approved by the local ethics committee (date of approval: 13 August 2010; assigned ID-number: 415-E/1122/13-2010) as well as by all participating centers. An undersigned informed consent was given from all patients.

### 2.2. Trial Design and Hypotheses, Definition of Primary and Secondary Endpoints

In a one-armed prospective multicenter trial, the 5-year IBR-rate in 3 different age groups (35–40, 41–50, >50 y) was tested against respective lowest recurrence rates from prospective RCTs published until 2010 and defined as primary study endpoint. Accordingly, benchmarks for superiority were defined as 2% for patient age above 50 y [25], 3.6% for patients between 41–50 and 35–40 y [26]. In contrast if 5-year-recurrence rates would exceed 3.5% (>50 y), 6% (41–50 y), and 10% (35–40 y) [27] inferiority would be stated, respectively (Supplementary Figures S1–S3). The sequential ratio test (SQRT) [28] was used to prove the hypothesis. Disease-free survival (DFS), as event due to breast cancer comprising local/regional recurrences, metastases and death from disease, metastases free survival (MFS) for any distant relapse, disease specific survival (DSS) for deaths due to breast cancer only, overall survival (OS) for deaths due to any reason, overall local control (LC) for IBR, and overall locoregional control (LRC) for IBR together with events in ipsilateral regional lymph nodes were defined as secondary endpoints. Furthermore, acute/late toxicity and cosmetic outcome (CO) (both patient-reported/subjective and physician-reported/objective as supported by standardized photo documentation) were evaluated by valid international scores [29,30,31,32] (Supplementary Tables S2 and S3) [24].

### 2.3. Treatment Schedule

After tumor removal by BCS (lumpectomy or oncoplastic surgery (OPS I-II [33,34]), the approximated tumorbed received an IOERT boost of 11.1 Gy (Dmax). The technical principle of IOERT was published previously in recent European practical guidelines, including considerations on appropriate target volumes and dosage for a boost concept [10]. Axillary lymph node dissection followed the sentinel node concept [35], which considered no further lymph node exploration for negative nodes or sentinel micrometastases [36] (protocol amendment 14 September 2011). When wound healing was completed, HWBI of 40.5 Gy was administered in 15 fractions (2.7 Gy single dose), considering a time gap to surgery of 6–8 weeks up to 9 months if adjuvant chemotherapy (CTX) was given. HWBI was delivered in supine position as 3D conformal radiation therapy (using 6/15 MV photons) with tangential fields or IMRT when appropriate. V20 thresholds for the ipsilateral lung <20% and <5% for the heart, were set as obligatory dose constraints, respectively. Supplementary Table S4 gives a detailed overview about technical parameters for IOERT and HWBI. Systemic treatment was mainly delivered on recommendations of the St. Gallen Consensus Conference since 2013 [37].

### 2.4. Data Registration, Quality Assurance, and Follow-Up

Patient follow-up started in week 4 and continued at month 4–5, year one, and annually thereafter. For clinical data collection (including photo-documentation), a central electronic database was established. In order to ensure quality assurance (QA), a centralized monitoring of the treatment plans was performed.

## 3. Statistical Methods

**Figure 1:**
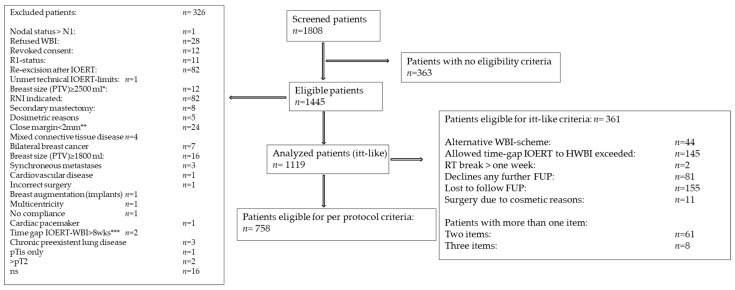
CONSORT-diagram: Study-patient acquisition. * Protocol amendment 21 August 2015: The upper limit breast volume was opened from 1800 to 2500 mL. ** Protocol amendment 21 August 2015: Clear margins were adapted to “no ink an tumor” *** patients have been considered for “itt analysis” since 27 September 2014. RNI: regional node irradiation; WBI: whole breast irradiation; ns: not stated; itt: intention to treat, FUP: follow-up, HWBI: hypofractionated whole breast irradiation.

Data were checked for consistency and screened for outliers. The sequential ratio test (SQRT) was used to test the hypotheses for the primary endpoint. This dynamic statistical model demands neither a previously determined fixed sample size nor a determination of accrual periods. SQRT typically needs lower expected sample sizes than designs with fixed ones. Power and sample size computations were done to achieve a power of 90% to demonstrate inferiority (i.e., five-year IBR-rates >10%, >6%, and >3.5% in each age group (35–40, 41–50, and >50 y, respectively). The earliest time point for a decision in favor of superiority (i.e., five-year IBR-rates of ≤3.6%, ≤3.6%, and ≤2%) occurs when the first 33, 90, and 146 patients within the respective age groups have completed the five-year follow-up free from local recurrence. The four-year rates for LC, LRC, DFS, MFS, DSS, and OS including its 95% confidence intervals (CI) were calculated using the Kaplan–Meier method. Statistical analysis was performed by intention to treat (itt) and named as “itt-like” due to the one-armed trial design. Itt-like criteria are depicted in Figure 1. Cumulative incidence curves with 95% confidence intervals of risks and occurrences of in-breast recurrences were computed to illustrate these results over time. All statistical tests were performed as one-sided, with *p*-values of <0.05 for significance. Calculations were done with STATISTICA 13 (Hill, T. & Lewicki, P. Statistics: Methods and Applications. StatSoft, Tulsa, OK, USA), Wolfram Research, Inc., Mathematica, Version 11.3, Champaign, IL, USA, (2020), and PASW 26 (IBM SPSS Statistics for Windows, Version 26.0., Armonk, NY, USA).

## 4. Results

As of April 2020, 1445 patients were identified as eligible for the trial (Figure 1) by eighteen active institutions. Out of these, 326 patients were excluded, leaving 1119 with a median age of 58 years (range 35–87) to be analyzed.

### 4.1. Primary Endpoint and Systemic Treatment

**Figure 2:**
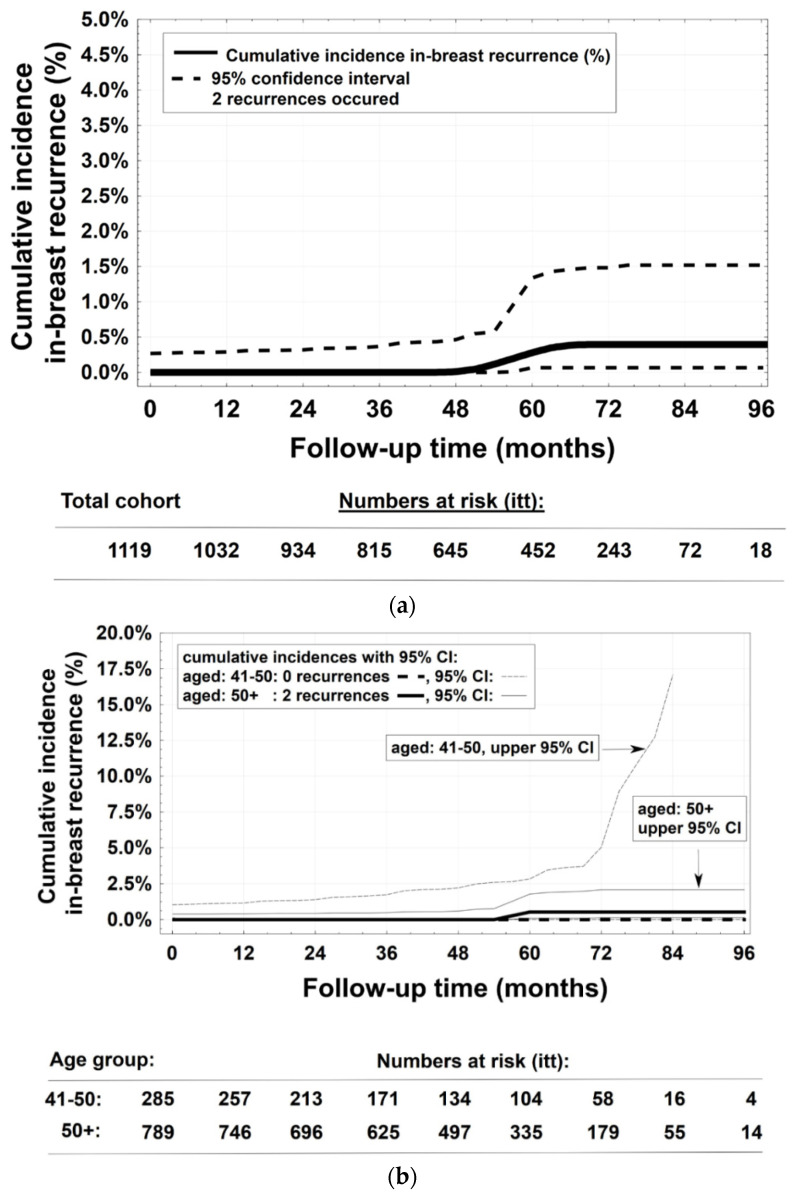
(**a**) Cumulative incidences of in-breast recurrences, whole study cohort. (**b**) Cumulative incidences of in-breast recurrences, age groups 50+ and 41–50.

CTX (primarily taxane and anthracycline containing regimens) was administered in 24% of patients either in adjuvant (19%) or neoadjuvant (5%) order. In the case of a positive Her2/neu status, trastuzumab ± pertuzumab was applied (overall 5.5%, alone or combined with CTX). Moreover, 88% of all patients were treated with endocrine therapy (ET), in 16% together with CTX. After a median follow-up time of 50 months (0.7–104), two IBR were noted in the group >50 years of age (*n* = 789) and none for the groups 41–50 y (*n* = 285) and 35–40 y (*n* = 45), respectively (Figure 2a,b). For each age group, the five-year patient accrual was depicted per protocol (pp) and intention to treat (itt) in Supplementary Figures S1–S3 as numbers at risk. By means of the SQRT, the expected best benchmarks of five-year IBR-rates in determined age groups were surpassed in age groups >50 after 158 patients (in May 2018) and in the age cohort 41–50 years after 92 patients (in October 2019) as no IBR was detected up to then (Supplementary Figures S1 and S2). Although no IBR was detected for patients 35–40 years of age, no statistical decision is yet possible due to low recruitment of only 11 patients in year five (target value *n* = 33). In this patient group, sampling is still ongoing (Supplementary Figure S3).

### 4.2. Secondary Endpoints

In the total cohort, 25 patients died (six due to breast cancer), 23 have metastasized, and one developed a regional supraclavicular relapse (Supplementary Table S5). The respective actuarial four-year rates for, DFS, MFS, DSS, OS, LC, and LRC were found to be 97.8% (95% CI 96.9–98.8), 98.1% (95% CI 97.2–99), 99.4% (95% CI 98.8–99.9), 97.9% (95% CI 96.6–98.9), 100% (95% CI 100), and 99.7% (95% CI 99.4–100).

Perioperatively, 65 (5.8%) major complications were noted, which were summarized in Supplementary Table S6. Early toxicity was classified as CTCAE G0/1 in 99.7 % (end of WBI) and 99.8 % (week 4) (Table 2). CTCAE G3 and G4 were observed in two patients and reported previously [24].

LENT-SOMA-ratings for late reactions (mean values, ranges) were performed at four to five months, 12 months, and annually thereafter until year nine. By taking into account all respective follow-ups and dependent on type of sequelae, G0/2 was quantified in 99.6% (99.3–100) and G3/4 reactions in 0.3% (0–1.9) of patients. (Table 2; Pain characteristics (classified as G4) of three patients, are explained and listed in Supplementary Table S6).

Baseline cosmesis was rated as at least satisfactory in 86% (patient-reported) and 76% (physician-reported) of patients, as acceptable in 98% (patient- as well as physician-reported) and as bad in 2% (patient- as well as physician-reported). The respective mean patient- and physician-reported satisfactory ratings were 78% (range 0–95), and 67% (range 0–87) at 4–5 and 12 months post HWBI and the annual follow-ups thereafter. Bad cosmesis (unacceptable results) was determined in only 1.9% (range 0–2) by patients and 4% (range 0–8) by physicians with no observed complications (Supplementary Figures S4 and S5 and Supplementary Table S7).

Of note, 11 patients underwent a second surgery for cosmetic reasons during the follow-up period. They were considered for the current analysis per intention to treat and quoted in Supplementary Table S6, respectively.

## 5. Discussion

In the last decade, IOERT with single doses of 10–11 Gy has been well established among breast boost techniques, as summarized in current European treatment guidelines [10]. In comparison to postoperative boost techniques, IOERT completely spares the skin and does not lead to volume distensions by (hemato-)seroma [38,39] resulting in a marked decrease in target volumes sizes, which causes no higher recurrence risk, but a better cosmetic long-term outcome [23]. Furthermore, when compared to external electron boosts, IOERT seems to prolong the time span to in-breast relapses remarkably [23]. Within the scope of the current literature for IOERT as boost followed by conventional WBI (single does 1.8–2 Gy up to total dosages of 50–54 Gy) local recurrence rates of 0.8 and 2.7% were reported after a median FUP of six and ten years, respectively [10]. Similar results were observed for IORT with 50-kv photons (21 Gy surface dosage) plus 46–50 Gy WBI (1.8–2 Gy/fx) with actuarial five-year rates of 1.7–2% after median observation times between three to six years [12,40,41,42,43]. Of note, in terms of late effects, boost-IORT with photons seems to initiate more likely higher fibrosis of grading III (three-year rate: 4–5%; five-year-rate 4–5%) [12,42,44] than observed in the present trial (three-year-rate: 1%; five-year-rate: 0.9%).

As early as 1989, Fowler et al. proposed that the α/β ratio of breast cancer might be as low as around four [45], which prompted prospective trials randomizing moderate HWBI (39–40 Gy/three weeks) against the established standard WBI (50 Gy/five weeks). Within these studies, non-inferiority was demonstrated for the experimental arms in terms of treatment tolerance as well as oncological outcome and was confirmed by a meta-analysis in 2016 [1]. For the experimental groups, overall IBR-rates at five and ten years were reported as high as 2.8 [26], 2.2 [25], 3.5–5.2 [46], and 3.8–8.1% [47,48], respectively. Analyzed by age, the Canadian and the UK START-B study-groups described the lowest five-year IBR- rates (age groups <40 y: 3.6% [26], 41–50 y: 3.6% [26], and >50 y: 2% [25]), which were benchmarked first by the EORTC trialist group for age groups <40 y with 10%, 41–50 y with 6%, and >50 y with 3.5% in 2001 [27]. However, within these pioneering trials, the potential of a tumor bed boost to further reduce IBR rates was not systematically investigated.

Therefore, our experimental design tested the possible gain by an 11 Gy IOERT boost (bioequivalent to 27.5 Gy (EQD2) considering an α/β value of 4) followed by HWBI along the START-B concept [25] within three age groups. Within the given statistical model, superiority was confirmed for patients in the age groups >50 years as well as 41–50 years. However, despite no detected IBR, no statistical statement was possible for the youngest age group due to insufficient patient accrual.

Of patients >50 years (*n* = 789), 26% (*n* = 209) showed at least one risk factor motivating a tumor bed boost according to current guidelines (negative HR-status, positive Her2neu-status, KI67 ≥30%, tumor size ≥2 cm, multifocality, pN1/× and EIC+). Of note, the two patients developing an in-breast relapse would be considered as biologically low-risk, with age >50 y, luminal A subtype and R0-resection (3–4 mm margin width). Recurrences were detected 4–4.5 y after HWBI. However, one patient had a tumor size ≥2 cm, and the other positive nodes, both factors described as possible negative predictors for IBRs [49,50]. Although rather rare events, this underlines once more the necessity of long-term follow-ups also for patients deemed to be at low risk for recurrence. Overall, acute and late treatment toxicity were mild, with very satisfactory cosmetic outcomes over time, which aligned with respective data ranges of previous reports [24,51].

### Trial Limitations

Despite a phase III design of an SQRT, the reported results are not based on a conventionally randomized approach. Furthermore, the outcomes were compared to historical patient cohorts of phase III trials, which reported the best results at the start point of the present study. Since then, and apart from progress in endocrine treatment and CTX, novel drugs like targeted antibodies (e.g., Trastuzumab and Pertuzumab) and immunomodulators like checkpoint-inhibitors [52] have been introduced. These developments may have additional implications for local control. Moreover, with the aim of deescalating local therapies, about 51% of our cohort would today be deemed as “suitable candidates” for PBI only [53,54], either with IOERT, external photons, multicatheter brachytherapy [55], or protons [56,57,58], respectively.

## 6. Conclusions

The combination of IOERT as tumorbed–boost followed by HWBI was superior to five-year IBR rates of best published phase III trials in patients >50 as well as 41–50 y of age after BCS. Promising results were also seen for patients in the age group 35–40 y, although no decision on inferiority or superiority was possible so far. Therefore, within reported radiation concepts for WBI plus boost, the HIOB strategy compares favorably in terms of local control rates, while providing mild toxicity and good cosmetic outcome.

## Figures and Tables

**Table 1:** Patient characteristics.

Characteristics	*n* (%)	Characteristics	*n* (%)
histology		pathological tumorstage	
IDC	656 (59)	T1	918 (82)
NST	214 (19)	T2	143 (13)
ILC	103 (9)	Tx	5 (0.5)
mixed	88 (8)	pathological nodalstage	
others	58 (5)	N0	934 (83)
EIC pos		N1	129 (12)
yes	149 (13)	Nx	3 (0.5)
no	970 (87)	y pathological tumorstage (NACT)	
grading		T0	20 (1)
G1	268 (24)	T1	31 (3)
G2	629 (56)	T2	2 (0.5)
G3	168 (15)	y pathological nodalstage (NACT)	
Gx	54 (5)	N0	50 (3.9)
Her2/neu status		N1	1 (0.1)
pos	157 (14)	Nx	2 (0.5)
neg	961 (85.9)	pCR	
ns	1 (0.1)	yes	29 (55)
HR-Status		no	24 (45)
pos	1020 (91)	Multifocality	
neg	98 (8.9)	Yes	140 (13)
ns	1 (0.1)	No	979 (87)
KI67 (%)		systemic treatment	
<20%	487 (44)	ET	983 (88)
≥20%	494 (44)	Adj. CTX and/or Tra ± Per	213 (19)
ns	138 (12)	NACT ± Tra ± Per	53 (5)
Age groups (y)		ET/CTX	183 (16)
35–40	45 (4)	Tra/+-Per	61 (5.5)
41–50	285 (26)	Resection margins	Median (range)
>50	789 (70)	distance (mm)	5 (0.1–80)
		ns	31 (3)

IDC: Invasive ductal; NST: no special type; ILC: Invasive lobular; others: tubular, medullary, mucinous, metaplastic; EIC-comp.: extensive intraductal component; HR: Hormonal receptor; ET: endocrine therapy; CTX: Chemotherapy; adj.: adjuvant; NACT: Neoadjuvant CTX, Tra; Trastuzumab; Per: Pertuzumab, y: years.

**Table 2:** (**a**) Acute and late toxicity (pain, breast edema, fibrosis). (**b**) Late toxicity (teleangiectasia, arm lymphedema, retraction/atrophy).

**(a)**										
**Acute Toxicity Grade** **RTOG-CTCAE Vers. 2 ^Ω^**	***n* = 1118: End of WBI** **% of eP** ***n* = 1097**	***n* = 1103: w 4** **% of eP:** ***n* = 1042**								
0	11	37.2								
1	80	56.2								
2	8.7	6.4								
3–4	0.3	0.2								
ns (%)	1.9	5.5								
**Late Toxicicty Grade** **LENT SOMA Scale ^Ω^**	**m 4/5** ***n* = 1091**	**y 1** ***n* = 1049**	**y 2** ***n* = 958**	**y 3** ***n* = 863**	**y 4** ***n* = 692**	**y 5** ***n* = 518**	**y 6** ***n* = 348**	**y 7** ***n* = 98**	**y 8** ***n* = 33**	**y 9** ***n* = 1**
Pain	% of eP: *n* = 1033	% of eP: *n* = 1000	% of eP: *n* = 907	% of eP: *n* = 828	% of eP: *n* = 664	% of eP: *n* = 497	% of eP: *n* = 343	% of eP: *n* = 97	% of eP: *n* = 29	*n* = 1
0	65.6	74	77.4	80.4	79.6	81.5	82.2	86.6	82.7	100
1	25.8	22.4	18.3	16.4	17.1	15	12.2	12.4	13.8	0
2	8	3.1	3.9	2.9	2.8	2.6	5.3	1	3.5	0
3–4	0.6	0.5	0.4	0.3	0.5	0.9	0.3	0	0	0
ns (%)	58 (5)	49 (5)	51 (5)	35 (4)	28 (4)	21 (4)	5 (1)	2 (2)	4 (12)	0
Breast edema	% of eP: *n* = 1031	% of eP: *n* = 998	% of eP: *n* = 908	% of eP: *n* = 828	% of eP: *n* = 660	% of eP: *n* = 498	% of eP: *n* = 342	% of eP: *n* = 97	% of eP: *n* = 29	*n* = 1
0	75	85.7	91	95.2	95.3	96.2	95.9	97.9	100	100
1	22.4	12.4	8.1	4.5	4.2	3.8	3.8	2.1	0	0
2	2.6	1.9	0.9	0.3	0.5	0	0.3	0	0	0
3	0	0	0	0	0	0	0	0	0	0
ns (%)	60 (5)	51 (5)	50 (5)	35 (4)	32 (5)	20 (4)	6 (2)	2 (2)	4 (12)	0
Fibrosis	% of eP: *n* = 1030	% of eP: *n* = 1002	% of eP: *n* = 908	% of eP: *n* = 828	% of eP: *n* = 660	% of eP: *n* = 498	% of eP: *n* = 343	% of eP: *n* = 97	% of eP: *n* = 29	*n* = 1
0	59	59.3	59.8	60.6	61.3	59.8	56.3	53.6	51.7	0
1	32.9	34.3	33.4	32.1	30.3	31.1	32.9	37.1	34,5	100
2	7.3	5.6	5.8	6.3	7.6	8.2	9.9	9.3	13.8	0
3	0.8	0.8	1	1	0.8	0.9	0.9	0	0	0
ns (%)	61(5)	47 (4)	50 (5)	35 (4)	32 (5)	20 (4)	5 (1)	2 (2)	4 (12)	0
**(b)**										
**Late Toxicicty Grade** **LENT SOMA Scale ^Ω^**	**m 4/5** ***n* = 1091**	**y 1** ***n* = 1049**	**y 2** ***n* = 958**	**y 3** ***n* = 863**	**y 4** ***n* = 692**	**y 5** ***n* = 518**	**y 6** ***n* = 348**	**y 7** ***n* = 98**	**y 8** ***n* = 33**	**y 9** ***n* = 1**
Teleangiectasia	% of eP: *n* = 1030	% of eP: *n* = 996	% of eP: *n* = 904	% of eP: *n* = 822	% of eP: *n* = 661	% of eP: *n* = 497	% of eP: *n* = 343	% of eP: *n* = 97	% of eP: *n* = 29	*n* = 1
0	96.2	95.9	95	94	94	94.2	91.8	92.8	89.6	100
1	3.1	3.6	3.3	3.5	3.6	2.8	5.5	4.1	10.4	0
2	0.6	0.5	1.7	2.4	2.2	2.8	2.1	3.1	0	0
3	0.1	0	0	0.1	0.2	0.2	0.6	0	0	0
ns (%)	61(5)	53 (5)	54 (6)	41 (5)	31 (4)	21 (4)	5 (1)	2 (2)	4 (12)	0
Arm lymphedema	% of eP: *n* = 1026	% of eP: *n* = 989	% of eP: *n* = 900	% of eP: *n* = 823	% of eP: *n* = 657	% of eP: *n* = 497	% of eP: *n* = 343	% of eP: *n* = 97	% of eP: *n* = 29	*n* = 1
0	98.6	97.9	98.4	99.4	99.4	99.5	99.8	99	96.5	100
1	1.4	2	1.3	0.6	0.6	0.5	0.2	1	3.5	0
2	0	0.1	0.3	0	0	0	0	0	0	0
3–4	0	0	0	0	0	0	0	0	0	0
ns (%)	65 (6)	60 (6)	58 (6)	40 (5)	35 (5)	21 (4)	5 (1)	2 (2)	4 (12)	0
Retraction/atrophy	% of eP: *n* = 1030	% of eP: *n* = 994	% of eP: *n* = 904	% of eP: *n* = 818	% of eP: *n* = 661	% of eP: *n* = 498	% of eP: *n* = 342	% of eP: *n* = 97	% of eP: *n* = 29	*n* = 1
0	81.7	75.5	75.1	73.2	70.6	63.6	60.8	47.4	51.7	0
1	16.7	21.5	21.3	23.3	25.5	30.7	33.6	50.5	44.8	100
2	1.2	2.3	2.8	2.6	2.3	3.8	4.1	2.1	3.5	0
3–4	0.4	0.7	0.8	0.9	1.6	1.9	1.5	0	0	0
ns (%)	61 (6)	55 (5)	54 (6)	45 (5)	31 (4)	20 (4)	6 (2)	2 (2)	4 (12)	0

Ω: Clinical interpretation for Grading 0–IV were summarized in supplementary Table S2, w: week, m: month, y: year, eP: evaluated patients, ns: not stated.

## Data Availability

First data on toxicity and cosmetic outcome were published in 2021 [24]. The source data for the analysis presented in this manuscript are available on request from the corresponding author.

## Supplementary Materials

The following supporting information can be downloaded at: https://www.mdpi.com/article/10.3390/cancers14061396/s1, Figure S1: 5-year local recurrence rate: SPRT for age group > 50 y, Figure S2: 5-year local recurrence rate: SPRT for age group 41–50 y, Figure S3: 5-year local recurrence rate: SPRT for age group 35–40 y, Figure S4: Subjective cosmesis over time (ratings patient-reported), Figure S5: Objective cosmesis over time (ratings physician-reported), Table S1: Exclusion criteria, Table S2: RTOG CTC-Score version 2 and LENT SOMA scale, Table S3: Cosmesis Score, Table S4: Technical parameters, Table S5: Overview of the clinical status, Table S6: Perioperative major complications (PMC), LENTSOMA G4 pain, second surgery for cosmetic reasons, Table S7: Cosmesis evaluation

## Abbreviations

IOERT	intraoperative radiation with electrons
HWBI	hypofractionated whole breast irradiation
BCS	breast conserving surgery
CO	cosmetic outcome
IBR	in breast recurrence
DCIS	ductal carcinoma in situ
RCTs	randomized controlled trials
HIOB	hypofractionated whole breast irradiation preceded by an intraoperative tumorbed boost with electrons
SQRT	sequential ratio test
FUP	follow-up
WBI	whole breast irradiation
RNI	regional node irradiation
PTV	Planning Target Volume
DFS	disease free survival
MFS	metastases free survival
DSS	disease specific survival
OS	overall survival
LC	overall local control
LRC	locoregional control
OPS	oncoplastic surgery
IMRT	intensity modulated radiotherapy
V20	tissue volume which receives 20 Gy or more
QA	quality assurance
ISIORT	International Society of Intraoperative Radiotherapy
CI	confidence interval
itt	intention to treat
pp	per protocol
ET	endocrine therapy
CTX	chemotherapy
H0	Null hypothesis

## Appendix A

**Table A1:** Membership of the HIOB Trialist Group.

Thorsten Fischer ^1^	Karin Dagn ^2^
Beata Adamczyk ^3^	Christoph Fussl ^2^
Piotr Milecki ^8^	Sabine Gerum ^2^
Daniela di Cristino ^4^	Brane Grambozov ^2^
Kyle Arneson ^5^	Wolfgang Iglseder ^2^
Michaela Gruber ^6^	Julia Kaiser ^2^
Anna Schiattarella ^7^	Josef Karner ^2^
Vincenzo Fusco ^9^	Andrea Kopp ^2^
Marina Alessandro ^10^	Michael Kopp ^2^
Antonio Stefanelli ^11^	Matthias Mattke ^2^
Elisabeth Bräutigam ^12^	Falk Röder ^2^
Giovanni Battista Ivaldi ^13^	Stefanie Windischbauer ^2^
Karl-Axel Hartmann ^14^	Frank Wolf ^2^
Marco Krengli ^15^	Franz Zehentmayr ^2^
Umberto Ricardi ^16^	

1. Department of Gynecology, Paracelsus Medical University, University Hospital Salzburg, Landeskrankenhaus, Salzburg, Austria

2. Department of Radiotherapy and Radio-Oncology, Paracelsus Medical University, University Hospital Salzburg, Landeskrankenhaus, Salzburg, Austria

3. Department of Surgical Oncology, Greater Poland Cancer Centre, Poznań, Poland

4. U.O.C. Radioterapia, San Filippo Neri Hospital, ASL Roma 1, Rome, Italy

5. Avera McKennan Hospitals and University Health System, Avera Medical Group, Comprehensive Breast Care, Sioux Falls, SD, USA

6. Department of Radiotherapy and Radiooncology, Landeskrankenhaus Klagenfurt, Klagenfurt, Austria

7. Department of Radiation Oncology, Azienda Sanitaria Universitaria Giuliano-Isontina, Trieste, Italy

8. Department of Radiotherapy Greater Poland Cancer Center and Chair of Electroradiology Poznan University of Medical Sciences, Poznań, Poland

9. Radiotherapy Department, IRCCS-CROB Reference Cancer Center Basilicata, Rionero in Vulture, Italy

10. Division of Radiation Oncology, Ospedale di Città di Castello, USL UMBRIA 1, Città di Castello, Italy

11. Azienda Ospedalio Universitaria Sant‘ Anna Ferrara, Ferrara, Italy

12. Department of Radiation Oncology, Ordensklinikum Linz Barmherzige Schwestern, Linz, Austria

13. Department of Radiation Oncology, ICS Maugeri—IRCCS, Pavia, Italy

14. Klinik für Strahlentherapie und Radiologische Onkologie, Marien Hospital Düsseldorf GmbH, Düsseldorf, Germany

15. Department of Radiation Oncology, University Hospital Maggiore della Carità, Novara, Italy

16. Department of Oncology, University of Turin, Turin, Italy

## References

[1] James M.L., Lehman M., Hider P.N., Jeffery M., Hickey B.E., Francis D.P. (2016). Fraction size in radiation therapy for breast conservation in early breast cancer. Cochrane Database Syst. Rev..

[2] Antonini N., Jones H., Horiot J.C., Poortmans P., Struikmans H., Van den Bogaert W., Barillot I., Fourquet A., Jager J., Hoogenraad W. (2007). Effect of age and radiation dose on local control after breast conserving treatment: EORTC trial 22881-10882. Radiother. Oncol..

[3] Bartelink H., Maingon P., Poortmans P., Weltens C., Fourquet A., Jager J., Schinagl D., Oei B., Rodenhuis C., Horiot J.C. (2015). Whole-breast irradiation with or without a boost for patients treated with breast-conserving surgery for early breast cancer: 20-year follow-up of a randomised phase 3 trial. Lancet Oncol..

[4] Cardoso F., Kyriakides S., Ohno S., Penault-Llorca F., Poortmans P., Rubio I.T., Zackrisson S., Senkus E. (2019). Early breast cancer: ESMO Clinical Practice Guidelines for diagnosis, treatment and follow-up. Ann. Oncol..

[5] Burstein H.J., Curigliano G., Loibl S., Dubsky P., Gnant M., Poortmans P., Colleoni M., Denkert C., Piccart-Gebhart M., Regan M. (2019). Estimating the benefits of therapy for early-stage breast cancer: The St. Gallen International Consensus Guidelines for the primary therapy of early breast cancer 2019. Ann. Oncol..

[6] Smith B.D., Bellon J.R., Blitzblau R., Freedman G., Haffty B., Hahn C., Halberg F., Hoffman K., Horst K., Moran J. (2018). Radiation therapy for the whole breast: Executive summary of an American Society for Radiation Oncology (ASTRO) evidence-based guideline. Pract. Radiat. Oncol..

[7] Woeckel A., Festl J., Stueber T., Brust K., Krockenberger M., Heuschmann P.U., Jírů-Hillmann S., Albert U.S., Budach W., Follmann M. (2018). Interdisciplinary Screening, Diagnosis, Therapy and Follow-up of Breast Cancer. Guideline of the DGGG and the DKG (S3-Level, AWMF Registry Number 032/045OL, December 2017)—Part 2 with Recommendations for the Therapy of Primary, Recurrent and Advanced Breast Cancer. Geburtshilfe Frauenheilkd..

[8] Brouwers P.J., van Werkhoven E., Bartelink H., Fourquet A., Lemanski C., van Loon J., Maduro J.H., Russell N.S., Scheijmans L.J., Schinagl D.A. (2018). Predictors for poor cosmetic outcome in patients with early stage breast cancer treated with breast conserving therapy: Results of the Young boost trial. Radiother. Oncol..

[9] Strnad V., Major T., Polgar C., Lotter M., Guinot J.L., Gutierrez-Miguelez C., Galalae R., Van Limbergen E., Guix B., Niehoff P. (2018). ESTRO-ACROP guideline: Interstitial multi-catheter breast brachytherapy as Accelerated Partial Breast Irradiation alone or as boost—GEC-ESTRO Breast Cancer Working Group practical recommendations. Radiother. Oncol..

[10] Fastner G., Gaisberger C., Kaiser J., Scherer P., Ciabattoni A., Petoukhova A., Sperk E., Poortmans P., Calvo F.A., Sedlmayer F. (2020). ESTRO IORT Task Force/ACROP recommendations for intraoperative radiation therapy with electrons (IOERT) in breast cancer. Radiother. Oncol..

[11] Wenz F., Blank E., Welzel G., Hofmann F., Astor D., Neumaier C., Herskind C., Gerhardt A., Suetterlin M., Kraus-Tiefenbacher U. (2012). Intraoperative radiotherapy during breast-conserving surgery using a miniature x-ray generator (Intrabeam®): Theoretical and experimental background and clinical experience. Women’s Health.

[12] Pez M., Keller A., Welzel G., Abo-Madyan Y., Ehmann M., Tuschy B., Berlit S., Sütterlin M., Wenz F., Giordano F.A. (2020). Long-term outcome after intraoperative radiotherapy as a boost in breast cancer. Strahlenther. Onkol..

[13] Sedlmayer F., Reitsamer R., Wenz F., Sperk E., Fussl C., Kaiser J., Ziegler I., Zehentmayr F., Deutschmann H., Kopp P. (2017). Intraoperative radiotherapy (IORT) as boost in breast cancer. Radiat. Oncol..

[14] Kaiser J., Kronberger C., Moder A., Kopp P., Wallner M., Reitsamer R., Fischer T., Fussl C., Zehentmayr F., Sedlmayer F. (2018). Intraoperative Tumor Bed Boost with Electrons in Breast Cancer of Clinical Stages I Through III: Updated 10-Year Results. Int. J. Radiat. Oncol. Biol. Phys..

[15] Belletti B., Vaidya J.S., D’Andrea S., Entschladen F., Roncadin M., Lovat F., Berton S., Perin T., Candiani E., Reccanello S. (2008). Targeted intraoperative radiotherapy impairs the stimulation of breast cancer cell proliferation and invasion caused by surgical wounding. Clin. Cancer Res..

[16] Veldwijk M.R., Neumaier C., Gerhardt A., Giordano F.A., Sütterlin M., Herskind C., Wenz F. (2015). Comparison of the proliferative and clonogenic growth capacity of wound fluid from breast cancer patients treated with and without intraoperative radiotherapy. Transl. Cancer Res..

[17] Herskind C., Wenz F. (2014). Radiobiological aspects of intraoperative tumour-bed irradiation with low-energy X-rays (LEX-IORT). Transl. Cancer Res..

[18] Sologuren I.R.-G.C., Lara P.D. (2014). Immune effects of high dose radiation treatment: Implications of ionizing radiation on the development of bystander and abscopal effects. Transl. Cancer Res..

[19] Kulcenty K., Piotrowski I., Wróblewska J.P., Wasiewicz J., Suchorska A.W.M. (2019). The Composition of Surgical Wound Fluids from Breast Cancer Patients is Affected by Intraoperative Radiotherapy Treatment and Depends on the Molecular Subtype of Breast Cancer. Cancers.

[20] Kulcenty K., Piotrowski I., Zaleska K., Wichtowski M., Wróblewska J., Murawa D., Suchorska W.M. (2019). Wound fluids collected postoperatively from patients with breast cancer induce epithelial to mesenchymal transition but intraoperative radiotherapy impairs this effect by activating the radiation-induced bystander effect. Sci. Rep..

[21] Kulcenty K.I., Piotrowski I., Zaleska K., Murawa D., Suchorska W.M. (2018). Wound fluids collected from patients after IORT treatment activates extrinsic apoptotic pathway in MCF7 breast cancer cell line. Ginekol. Polska.

[22] Reitsamer R., Sedlmayer F., Kopp M., Kametriser G., Menzel C., Deutschmann H., Nairz O., Hitzl W., Peintinger F. (2006). The Salzburg concept of intraoperative radiotherapy for breast cancer: Results and considerations. Int. J. Cancer.

[23] Ciabattoni A., Gregucci F., Fastner G., Cavuto S., Spera A., Drago S., Ziegler I., Mirri M.A., Consorti R., Sedlmayer F. (2021). IOERT versus external beam electrons for boost radiotherapy in stage I/II breast cancer: 10-year results of a phase III randomized study. Breast Cancer Res. BCR.

[24] Fastner G., Reitsamer R., Urbański B., Kopp P., Murawa D., Adamczyk B., Karzcewska A., Milecki P., Hager E., Reiland J. (2020). Toxicity and cosmetic outcome after hypofractionated whole breast irradiation and boost-IOERT in early stage breast cancer (HIOB): First results of a prospective multicenter trial (NCT01343459). Radiother. Oncol..

[25] Bentzen S.M., Agrawal R.K., Aird E.G., Barrett J.M., Barrett-Lee P.J., Bentzen S.M., Bliss J.M., Brown J., Dewar J.A., Dobbs H.J. (2008). The UK Standardisation of Breast Radiotherapy (START) Trial B of radiotherapy hypofractionation for treatment of early breast cancer: A randomised trial. Lancet.

[26] Whelan T., MacKenzie R., Julian J., Levine M., Shelley W., Grimard L., Lada B., Lukka H., Perera F., Fyles A. (2002). Randomized trial of breast irradiation schedules after lumpectomy for women with lymph node-negative breast cancer. J. Natl. Cancer Inst..

[27] Bartelink H., Horiot J.C., Poortmans P., Struikmans H., Van den Bogaert W., Barillot I., Fourquet A., Borger J., Jager J., Hoogenraad W. (2001). Recurrence rates after treatment of breast cancer with standard radiotherapy with or without additional radiation. N. Engl. J. Med..

[28] Wald A. (1945). Sequential Tests of Statistical Hypotheses. Ann. Math. Stat..

[29] Trotti A., Byhardt R., Stetz J., Gwede C., Corn B., Fu K., Gunderson L., McCormick B., Morris M., Rich T. (2000). Common toxicity criteria: Version 2.0. an improved reference for grading the acute effects of cancer treatment: Impact on radiotherapy. Int. J. Radiat. Oncol. Biol. Phys..

[30] Rubin P., Constine L.S., Fajardo L.F., Phillips T.L., Wasserman T.H. (1995). EORTC Late Effects Working Group. Overview of late effects normal tissues (LENT) scoring system. Radiother. Oncol..

[31] Pavy J.J., Denekamp J., Letschert J., Littbrand B., Mornex F., Bernier J., Gonzales-Gonzales D., Horiot J.C., Bolla M., Bartelink H. (1995). EORTC Late Effects Working Group. Late effects toxicity scoring: The SOMA scale. Radiother. Oncol..

[32] Van Limbergen E., van der Schueren E., Van Tongelen K. (1989). Cosmetic evaluation of breast conserving treatment for mammary cancer. 1. Proposal of a quantitative scoring system. Radiother. Oncol..

[33] Clough K.B., Kaufman G.J., Nos C., Buccimazza I., Sarfati I.M. (2010). Improving breast cancer surgery: A classification and quadrant per quadrant atlas for oncoplastic surgery. Ann. Surg. Oncol..

[34] McCulley S.J., Macmillan R.D. (2005). Therapeutic mammaplasty--analysis of 50 consecutive cases. Br. J. Plast. Surg..

[35] Krag D.N., Anderson S.J., Julian T.B., Brown A.M., Harlow S.P., Costantino J.P., Ashikaga T., Weaver D.L., Mamounas E.P., Jalovec L.M. (2010). Sentinel-lymph-node resection compared with conventional axillary-lymph-node dissection in clinically node-negative patients with breast cancer: Overall survival findings from the NSABP B-32 randomised phase 3 trial. Lancet Oncol..

[36] Giuliano A.E., Ballman K.V., McCall L., Beitsch P.D., Brennan M.B., Kelemen P.R., Ollila D.W., Hansen N.M., Whitworth P.W., Blumencranz P.W. (2017). Effect of Axillary Dissection vs No Axillary Dissection on 10-Year Overall Survival Among Women with Invasive Breast Cancer and Sentinel Node Metastasis: The ACOSOG Z0011 (Alliance) Randomized Clinical Trial. Jama.

[37] Goldhirsch A., Winer E.P., Coates A.S., Gelber R.D., Piccart-Gebhart M., Thürlimann B., Senn H.J., Albain K.S., André F., Bergh J. (2013). Personalizing the treatment of women with early breast cancer: Highlights of the St Gallen International Expert Consensus on the Primary Therapy of Early Breast Cancer 2013. Ann. Oncol..

[38] Hammer J., Mazeron J.J., Van Limbergen E. (1999). Breast boost—Why, how, when...?. Strahlenther. Onkol..

[39] Nairz O., Deutschmann H., Kopp M., Wurstbauer K., Kametriser G., Fastner G., Merz F., Reitsamer R., Menzel C., Sedlmayer F. (2006). A dosimetric comparison of IORT techniques in limited-stage breast cancer. Strahlenther. Onkol..

[40] Vaidya J.S., Baum M., Tobias J.S., Massarut S., Wenz F., Murphy O., Hilaris B., Houghton J., Saunders C., Corica T. (2006). Targeted intraoperative radiotherapy (TARGIT) yields very low recurrence rates when given as a boost. Int. J. Radiat. Oncol. Biol. Phys..

[41] Vaidya J.S., Baum M., Tobias J.S., Wenz F., Massarut S., Keshtgar M., Hilaris B., Saunders C., Williams N.R., Brew-Graves C. (2011). Long-term results of targeted intraoperative radiotherapy (Targit) boost during breast-conserving surgery. Int. J. Radiat. Oncol. Biol. Phys..

[42] Blank E., Kraus-Tiefenbacher U., Welzel G., Keller A., Bohrer M., Sütterlin M., Wenz F. (2010). Single-center long-term follow-up after intraoperative radiotherapy as a boost during breast-conserving surgery using low-kilovoltage x-rays. Ann. Surg. Oncol..

[43] Wenz F., Welzel G., Blank E., Hermann B., Steil V., Sütterlin M., Kraus-Tiefenbacher U. (2010). Intraoperative radiotherapy as a boost during breast-conserving surgery using low-kilovoltage X-rays: The first 5 years of experience with a novel approach. Int. J. Radiat. Oncol. Biol. Phys..

[44] Wenz F., Welzel G., Keller A., Blank E., Vorodi F., Herskind C., Tomé O., Sütterlin M., Kraus-Tiefenbacher U. (2008). Early initiation of external beam radiotherapy (EBRT) may increase the risk of long-term toxicity in patients undergoing intraoperative radiotherapy (IORT) as a boost for breast cancer. Breast.

[45] Fowler J.F. (1989). The linear-quadratic formula and progress in fractionated radiotherapy. Br. J. Radiol..

[46] Bentzen S.M., Agrawal R.K., Aird E.G., Barrett J.M., Barrett-Lee P.J., Bliss J.M., Brown J., Dewar J.A., Dobbs H.J., Haviland J.S. (2008). The UK Standardisation of Breast Radiotherapy (START) Trial A of radiotherapy hypofractionation for treatment of early breast cancer: A randomised trial. Lancet Oncol..

[47] Haviland J.S., Owen J.R., Dewar J.A., Agrawal R.K., Barrett J., Barrett-Lee P.J., Dobbs H.J., Hopwood P., Lawton P.A., Magee B.J. (2013). The UK Standardisation of Breast Radiotherapy (START) trials of radiotherapy hypofractionation for treatment of early breast cancer: 10-year follow-up results of two randomised controlled trials. Lancet Oncol..

[48] Whelan T.J., Pignol J.P., Levine M.N., Julian J.A., MacKenzie R., Parpia S., Shelley W., Grimard L., Bowen J., Lukka H. (2010). Long-term results of hypofractionated radiation therapy for breast cancer. N. Engl. J. Med..

[49] Polo A., Polgár C., Hannoun-Levi J.M., Guinot J.L., Gutierrez C., Galalae R., van Limbergen E., Strnad V. (2017). Risk factors and state-of-the-art indications for boost irradiation in invasive breast carcinoma. Brachytherapy.

[50] Truong P.T., Jones S.O., Kader H.A., Wai E.S., Speers C.H., Alexander A.S., Olivotto I.A. (2009). Patients with t1 to t2 breast cancer with one to three positive nodes have higher local and regional recurrence risks compared with node-negative patients after breast-conserving surgery and whole-breast radiotherapy. Int. J. Radiat. Oncol. Biol. Phys..

[51] Ivaldi G.B., Leonardi M.C., Orecchia R., Zerini D., Morra A., Galimberti V., Gatti G., Luini A., Veronesi P., Ciocca M. (2008). Preliminary results of electron intraoperative therapy boost and hypofractionated external beam radiotherapy after breast-conserving surgery in premenopausal women. Int. J. Radiat. Oncol. Biol. Phys..

[52] Dierks F., Pietsch E., Dunst J. (2020). Pembrolizumab as neoadjuvant treatment of early triple-negative breast cancer. Strahlenther. Onkol..

[53] Leonardi M.C., Maisonneuve P., Mastropasqua M.G., Morra A., Lazzari R., Rotmensz N., Sangalli C., Luini A., Veronesi U., Orecchia R. (2012). How do the ASTRO consensus statement guidelines for the application of accelerated partial breast irradiation fit intraoperative radiotherapy? A retrospective analysis of patients treated at the European Institute of Oncology. Int. J. Radiat. Oncol. Biol. Phys..

[54] Correa C., Harris E.E., Leonardi M.C., Smith B.D., Taghian A.G., Thompson A.M., White J., Harris J.R. (2017). Accelerated Partial Breast Irradiation: Executive summary for the update of an ASTRO Evidence-Based Consensus Statement. Pract. Radiat. Oncol..

[55] Strnad V., Krug D., Sedlmayer F., Piroth M.D., Budach W., Baumann R., Feyer P., Duma M.N., Haase W., Harms W. (2020). DEGRO practical guideline for partial-breast irradiation. Strahlenther. Onkol..

[56] Bush D.A., Do S., Lum S., Garberoglio C., Mirshahidi H., Patyal B., Grove R., Slater J.D. (2014). Partial breast radiation therapy with proton beam: 5-year results with cosmetic outcomes. Int. J. Radiat. Oncol. Biol. Phys..

[57] Pasalic D., Strom E.A., Allen P.K., Williamson T.D., Poenisch F., Amos R.A., Woodward W.A., Stauder M.C., Shaitelman S.F., Smith B.D. (2021). Proton Accelerated Partial Breast Irradiation: Clinical Outcomes at a Planned Interim Analysis of a Prospective Phase 2 Trial. Int. J. Radiat. Oncol. Biol. Phys..

[58] Mutter R.W., Choi J.I., Jimenez R.B., Kirova Y.M., Fagundes M., Haffty B.G., Amos R.A., Bradley J.A., Chen P.Y., Ding X. (2021). Proton Therapy for Breast Cancer: A Consensus Statement from the Particle Therapy Cooperative Group Breast Cancer Subcommittee. Int. J. Radiat. Oncol. Biol. Phys..

