# Supplementary material for: Hypofractionated Whole Breast Irradiation and Boost-IOERT in Early Stage Breast Cancer (HIOB): First Clinical Results of a Prospective Multicenter Trial (NCT01343459)
Source: Cancers (Basel). 2022 Mar 9;14(6):1396. doi: 10.3390/cancers14061396 (PMC8946807; doi:10.3390/cancers14061396)
Supplement: Supplementary file 1 [file cancers-14-01396-s001.zip › cancers-1574875-supplementary.pdf]

cancers

MDPI

Supplementary Materials: Hypofractionated Whole Breast Irradiation and Boost-IOERT in Early Stage Breast Cancer (HIOB): First Clinical Results of a Prospective Multicenter Trial (NCT01343459)

Gerd Fastner, Roland Reitsamer, Christoph Gaisberger, Wolfgang Hitzl, Bartosz Urbański Dawid Murawa, Christiane Matuschek, Wilfried Budach, Antonella Ciabattoni, Juliann Reiland, Marie Molnar, Cristiana Vidali, Claudia Schumacher, Felix Sedlmayer and on behalf of the HIOB Trialist Group

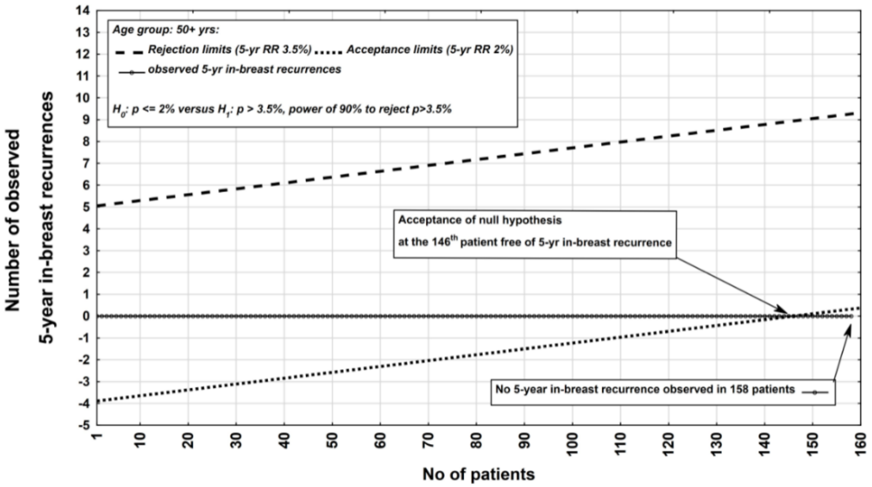

Numbers at risk

FUP, months Median, range	I, 1 1 (0.2-2.8)		II, 4-5 4.6 (2.8-8.7)		III, 13 12.9(7.9-20.9)		IV, 24 24.5 (18.8-32.8)		V, 36 36.5 (27.5-44.4)		VI, 48 48.4 (37.5-58.3)		**VII, 60 60.3(50.4-69.4)**		VIII, 72 72.3(63.8-82.7)		IX, 79 84.3(79.4-92.2)		X, 92 95.8(90-101.5)		XI, 108 102.8 (102-103.6)	
analysis	pp	itt	pp	itt	pp	itt	pp	itt	pp	itt	pp	itt	**pp**	**itt**	pp	itt	pp	itt	pp	itt	pp	itt
patients	657	123	650	122	636	120	597	108	559	100	462	75	**339**	**53**	225	34	59	16	16	9	0	1
sum patients	780		772		756		705		659		537		**392**		259		75		25		1	

Figure S1. 5-**year** local recurrence rate: SPRT for age group > 50 years. pp: per protocol, itt: intention to treat, FUP: follow-up.

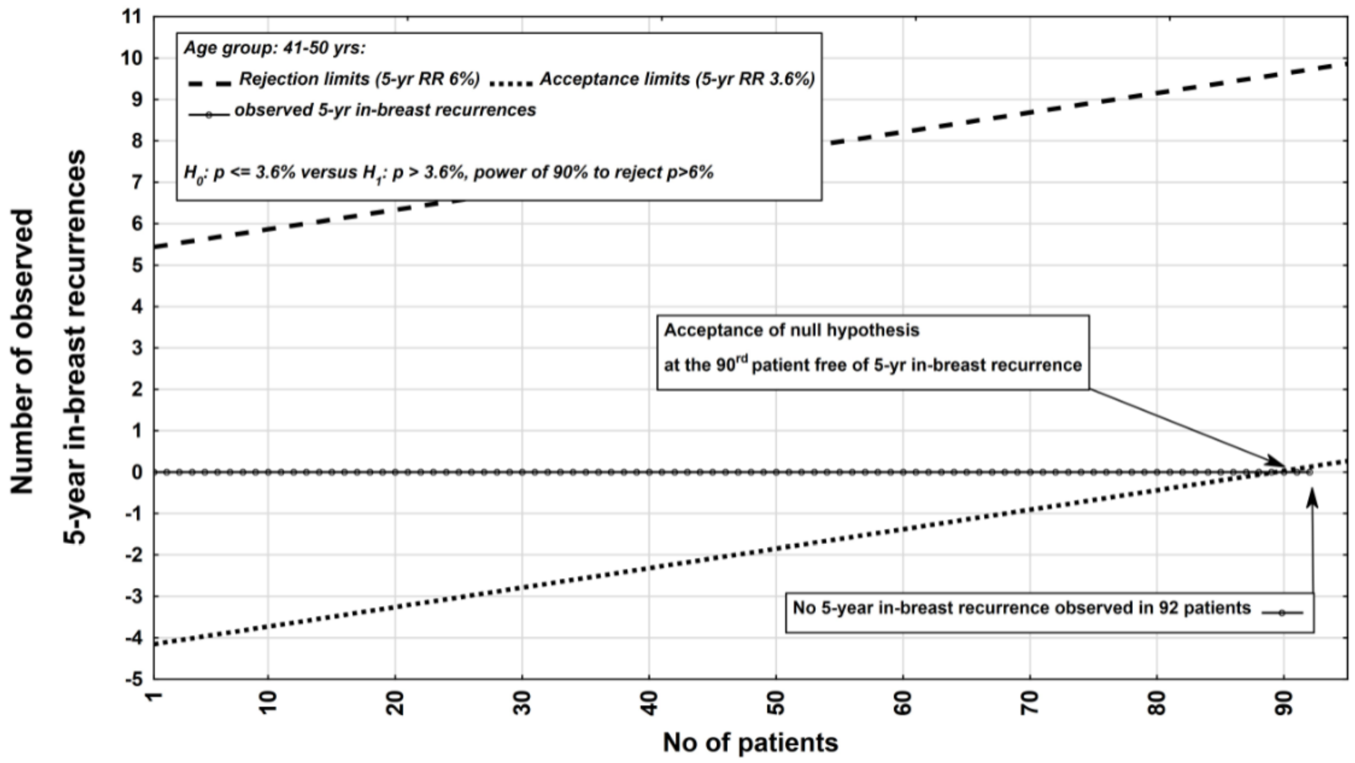

Numbers at risk

FUP, months	I, 1		II, 4-5		III, 13		IV, 24		V, 36		VI, 48		**VII, 60**		VIII, 72		IX, 84		X, 96		XI, 108	
Median, range	0.9 (0.3-2.7)		4.6 (2.9-8.6)		12.9(8.8-20.6)		24.4(18.3-32.2)		36.6 (29.3-45.6)		48.7 (41.1-57.6)		**60.5 (50.9-68.8)**		72.4 (68.7-80.2)		84 (80.8-87.5)		96.2 (93-96.8)		0	
analysis	pp	itt	pp	itt	pp	itt	pp	itt	pp	itt	pp	itt	**pp**	**itt**	pp	itt	pp	itt	pp	itt	pp	itt
patients	246	37	245	34	233	27	204	21	166	15	128	12	**104**	**9**	72	7	18	4	6	1	0	0
sum patients	283		279		260		225		181		140		**113**		79		22		7		0	

Figure S2. 5-year local recurrence rate: SPRT for age group 41-50 years. pp: per protocol, itt: intention to treat, FUP: follow-up.

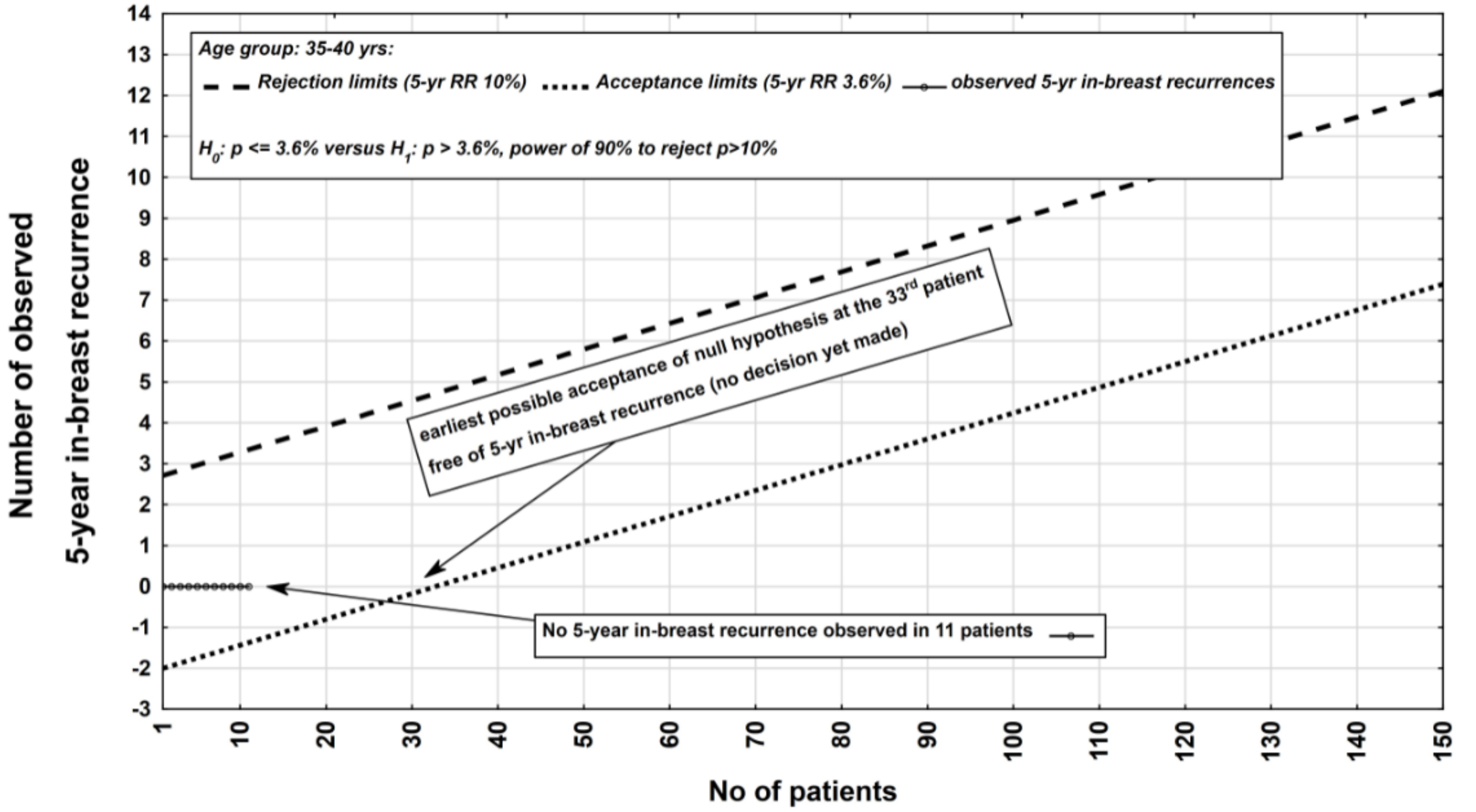

Numbers at risk

FUP, months	I, 1		II, 4-5		III, 13		IV, 24		V, 36		VI, 48		**VII, 60**		VIII, 72		IX, 84		X, 96		XI, 108	
Median, range	1(0.5-2.1)		4.7 (3.2-8)		13 (9.4-19.3)		25 (19.6-31.8)		36.9 (30.9-41)		48.9 (42.9-50.8)		**60.7 (55-63.3)**		72.9 (67-75.6)		79.5 (79.5-79.5)		92.3 (92.3-92.3)		0	
analysis	pp	itt	pp	itt	pp	itt	pp	itt	pp	itt	pp	itt	**pp**	**itt**	pp	itt	pp	itt	pp	itt	pp	itt
patients	36	4	36	4	29	4	24	4	20	3	13	2	**11**	**2**	9	1	1	0	1	0	0	0
sum patients	40		40		33		28		23		15		**13**		10		1		1		0	

Figure S3. 5-year local recurrence rate: SPRT for age group 35-40 years.

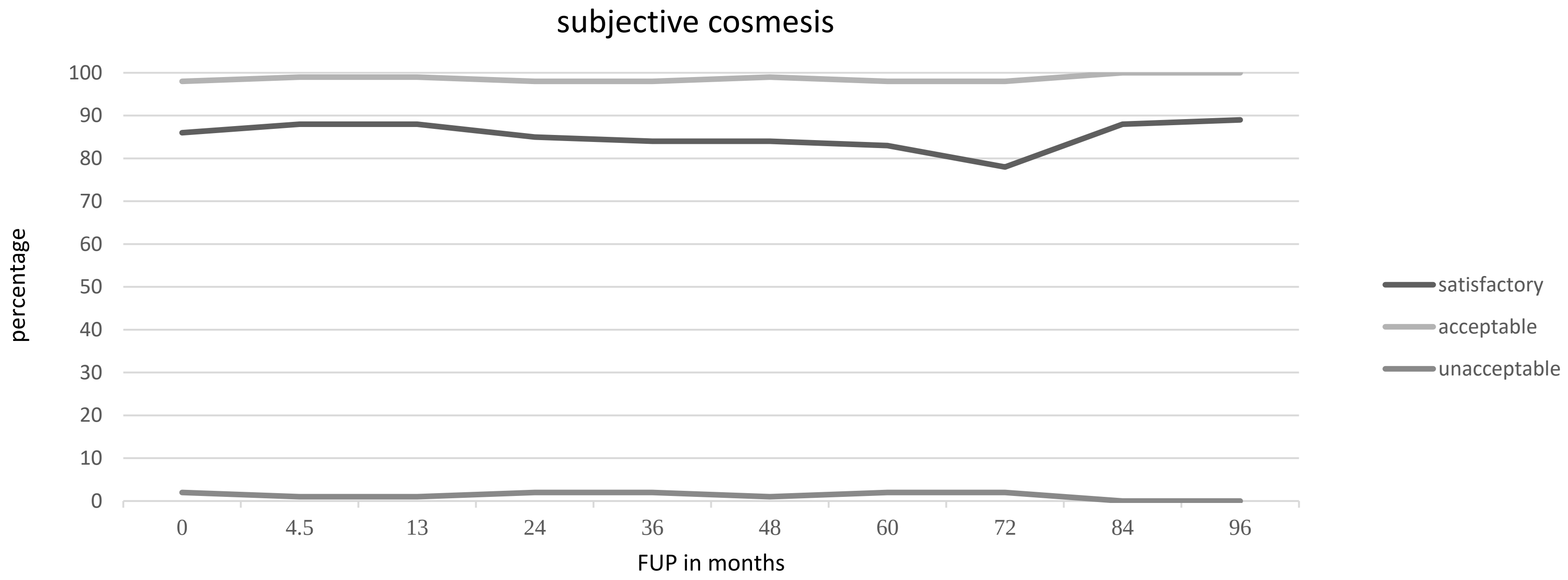

**Number at risk**	1118	1091	1049	958	863	692	518	348	98	33	1
**Median FUP months**		4.6	12.9	24.5	36.5	48.5	60.4	72.3	84.2	95.9	103.6
**Range FUP**		2.8-8.7	7.9-20.9	18.3-32.8	27.5-45.6	37.5-58.3	50.4-69.4	63.8-82.7	79.4-92.2	90-101.5	

Figure S4. Subjective cosmesis over time (ratings patient-reported).

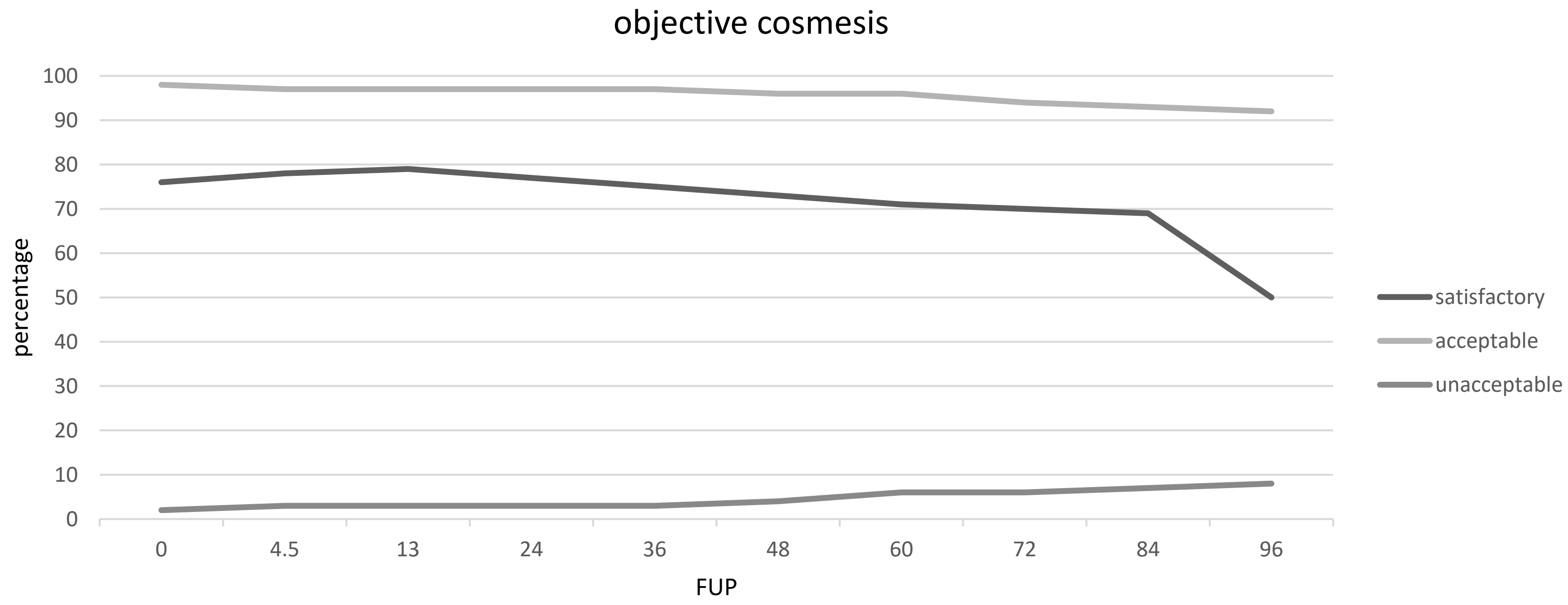

**Number at risk**	1118	1091	1049	958	863	692	518	348	98	33	1
**Median FUP months**		4.6	12.9	24.5	36.5	48.5	60.4	72.3	84.2	95.9	103.6
**Range FUP**		2.8-8.7	7.9-20.9	18.3-32.8	27.5-45.6	37.5-58.3	50.4-69.4	63.8-82.7	79.4-92.2	90-101.5	

Figure S5. Objective cosmesis over time (ratings physician-reported).

Table S1. Exclusion criteria.

Exclusion criteria
negation of inclusion criteria
in-situ carcinoma only
secondary mastectomy
previous RT to the affected breast
mixed connective tissue diseases
chronic pre-existent lung disease
cardiac co-morbidity
distant metastases
observed pregnancy
bilateral breast cancer
multicentricity
Karnofsky Index < 70%

RNI: regional node irradiation;.

Table S2. RTOG CTC-Score version 2 and LENT SOMA scale.

**Acute toxicity grade: RTOG-CTCAE Vers. 2 $^{\Omega}$**	***Clinical appearance***
0	None
1	Faint erythema, dry desquamation
2	Moderate to brisk erythema, moderate edema, moist desquamation (mostly confined to skin folds and creases)
3	Confluent moist desquamation > 1,5 cm and not confined to skin folds, pitting edema
4	Skin necrosis or ulceration of full thickness dermis; may include bleeding no induced by minor trauma or abrasion.
**Late toxicicty grade: LENT SOMA scale $^{\Psi}$**	
Pain	*Clinical appearance*
0	No
1	Occasional - minimal Hypersensation, Pruritus
2	Intermittend & tolerable
3	Persistent & intense
4	Refractory & excruciating
Breast edema	*Clinical appearance*
0	No
1	Asymptomatic
2	Symptomatic
3	Secondary dysfunction
Fibrosis	*Clinical appearance*
0	No
1	Barely palpable increased Density
2	Definite increased density and firmness
3	Very marked density, retraction and fixation
Teleangiectasia	*Clinical appearance*
0	No
1	<1/cm2
2	1/cm2 - 4/cm2
3	>4/cm2
Arm lymphedema	*Clinical appearance*
0	No

1	2 cm - 4 cm increase
2	>4 cm - 6 cm increase
3	>6 cm increase
4	2 cm - 4 cm increase
Retraction/atrophy	*Clinical appearance*
0	No
1	10%-25%
2	25% - 40%
3	40% - 75%
4	Whole breast
Ulcers	*Clinical appearance*
0	No
1	epidermal only,< 1 cm
2	Demal,> 1 cm2
3	Subcutaneous
4	Bone exposed,necrosis

Ω: reference [29] Ψ: reference [30,31].

Table S3. Cosmesis Score [Φ].

Single classification	*Cosmetic result*	*Clinical appearance*
E0	excellent	no visible therapy related sequelae
E1	good	minmal changes in pigmentation, visible scare, localized teleangiectasia
E2	moderate	clear deformation breast contur, nipple displacement, marked skin changes
E3	bad	Severe retraction or fibrosis, severe teleangiectasia
E4	complication	Skin necrosis

Summary classification	*Cosmetic result*
E0, E1	satisfactory
E2	moderate
E0,E1,E2	acceptable
E3,E4	unacceptable

Φ: reference [32].

Table S4. Technical parameters.

Technical parameters	
IOERT median (range)	
Tubediameter (cm)	6 (4-8)
Electron energy (MeV)	9 (4-15)
Mixed electron energy n (%)	128 (11)
Bevel angle (degree)	22 (0-45)
V90 (ml)	44 (4-104)
Depth calculation: n (%)	
CT	2 (0.2)
Probe/needle	331 (30)
ultrasound	774 (68.8)
ns	12 (1)
Lead-shielding: n (%)	
no	268 (24)
yes	851 (76)
HWBI	
Breast volume (PTV: ml)	1350 (233-2457)
ns (n)	4

MeV: Mega electron Voltage, V90: tissue volume encompassed by the IOERT 90% Isodose (10Gy),.ns: not stated, PTV: Planning target volume, ns: not stated.

Table S5. Overview of the clinical status.

Status	n (%)
DM	23 (2)
breast cancer	17 (1.5)
secondary cancers	6 (0.5)
Deaths	25 (2)
breast cancer	6 (0.5)
secondary cancers	8 (0.5)
other reasons	11 (1)
IBR	2 (0.2)
RR	1 (0.1)
Secondary cancer	49 (4)
Contralateral breast cancer	17 (1.5)
Others	32 (3)

DM: distant metastases, IBR: in-breast recurrences, RR: regional recurrence

Table S6. Perioperative major complications (PMJ), LENTSOMA G4 pain, second surgery for cosmetic reasons.

**PMJ**	**patients (n)**
hemorrhage	15
prolonged wound healing/disorders	18
seroma	10
wound infection/inflammation	7
no comments	15
**patient LENTSOMA G4 pain / point of FUP**	**comments**
patient 1 / months 4/5	accompanied with G3 fibrosis/shrinkage / declined any further FUP
patient 2 / year 3	developed metastases in year 2 / died in year 3 due to disease
patient 3 / months 4/5, year 1	G4 rating resolved in year 2 / lost to FUP thereafter
**second surgery for cosmetic reasons**	**patients (n)**
lipofilling	6
excision of liponecrosis	1
breast augmentation	1
breast reconstruction	2
reduction mastopexy	1

FUP: follow up.

Table S7. Cosmesis evaluation

Score	pre-WBI		FUP 4-5 months		FUP 12 months		FUP 24 months		FUP 36 months		FUP 48 months		FUP 60 months		FUP 72 months	
	subj n (%)	obj n (%)	subj n (%)	obj n (%)	subj n (%)	obj n (%)	subj n (%)	obj n (%)	subj n (%)	obj n (%)	subj n (%)	obj n (%)	subj n (%)	obj n (%)	subj n (%)	obj n (%)
E0	278(26)	160(17)	383(38)	240(26)	391(40)	264(29)	347(39)	240(29)	303(37)	220(29)	211(32)	178(30)	138(28)	125(28)	88(27)	81(26)
E1	631(60)	548(59)	513(50)	484(52)	469(48)	447(50)	412(46)	389(48)	378(47)	343(46)	333(52)	257(43)	269(55)	192(43)	171(51)	135(44)
E2	125(12)	207(22)	111(11)	174(19)	107(11)	163(18)	116(13)	162(20)	115(14)	160(22)	100(15)	137(23)	75(15)	101(23)	67(20)	72(24)
E3	14(2)	22(2)	12(1)	23(3)	12(0.9)	24(3)	10(2)	21(3)	10(2)	21(3)	6(1)	20(4)	8(2)	24(6)	6(2)	18(6)
E4					1(0.1)											
eP	1048	937	1019	921	980	898	885	812	806	744	650	592	490	442	332	306
ns	70 (6)	181 (16)	72 (6)	170 (15)	69 (6)	151 (14)	73 (7)	146 (15)	57 (7)	119 (14)	42 (6)	100 (14)	28 (5)	76 (15)	16 (4)	42 (12)

**Number at risk**	**1118**	**1091**	**1049**	**958**	**863**	**692**	**518**	**348**
**Median FUP (range)**		**4.6 (2.8-8.7)**	**12.9 (7.9-20.9)**	**24.5 (18.3-32.8)**	**36.5 (27.5-45.6)**	**48.5 (37.5-58.3)**	**60.4 (50.4-69.4)**	**72.3 (63.8-82.7)**

Score	FUP 84 months		FUP 96 months		FUP 108 months	
	subj n (%)	obj n (%)	subj n (%)	obj n (%)	subj n (%)	obj n (%)
E0	27(29)	18(20)	8(28)	5(21)		
E1	55(59)	44(49)	17(61)	7(29)		
E2	11(12)	21(24)	3(11)	10(42)	1(100)	1(100)
E3		6(7)		2(8)		
E4						
eP	93	89	28	24	1	1
ns	5 (2)	9 (9)	5 (15)	9 (27)	0	0

**Number at risk**	**98**	**33**	**1**
**Median FUP (range)**	**84.2 (79.4-92.2)**	**95.9 (90-101.5)**	**103.6**

eP: evaluated patients; ns: not stated; subj: subjective (i.e patient-reported); obj: objective (i.e physician-reported)
